# Cost of Hematopoietic Stem Cell Transplantation in India

**DOI:** 10.4084/MJHID.2014.046

**Published:** 2014-07-01

**Authors:** Sanjeev Kumar Sharma, Dharma Choudhary, Nitin Gupta, Mayank Dhamija, Vipin Khandelwal, Gaurav Kharya, Anil Handoo, Rasika Setia, Arpita Arora

**Affiliations:** Department of Hemato-oncology and Bone marrow transplantation. BLK Superspeciality Hospital, Pusa Road, New Delhi, India

## Abstract

Hematopoietic stem cell transplantation (HSCT) is a definite cure for many hematological diseases. With the increasing indications for HSCT and its relatively low cost in Indian subcontinent, an increasing number of patients are opting for this procedure. We retrospectively analyzed the cost of one hundred sixty two HSCTs done at our center in the last three years. The median cost of autologous transplant was USD, $ 12,500 (range $ 10,331–39,367) and the median cost of allogeneic transplant was $ 17,914 (range $ 10,832–44,701). The cost of HSCT is cheaper here compared to that in developed countries and success rates are nearly equivalent. The major factors contributing to the cost are related to the complications post-transplant mainly infections and graft versus host disease, which are also the reasons for the increased stay in the hospital.

## Introduction

One fifth of the world population resides in India. The burden of hematological diseases both malignant and non-malignant is huge in the country. About 10,000 children are born with thalassemia major each year, and about 6,000 cases are diagnosed with aplastic anemia per year.^1^,^2^ The number of leukemia and lymphoma patients is about 100,000.^3^ The number of patients requiring bone marrow transplant is also increasing. With the increasing awareness about hematological diseases and rising economy, many patients are opting for bone marrow transplant as a definite treatment for many curable hematological diseases. We retrospectively evaluated the cost of HSCT in our country and compared it with data from developed countries.

## Materials and Methods

### Study population

Between January 2011 and September 2013, a total of 162 patients with hematological diseases received HSCT at the Bone marrow transplant (BMT) center, BLK Superspeciality Hospital, New Delhi. The study included patients with thalassemia major, leukemia, lymphoma, aplastic anemia, multiple myeloma and others. Written informed consent for HSCT was provided by patients after a discussion of the risks and benefits of each method with the patient. The total cost included the cost of chemotherapy, stem cell/bone marrow harvest, antibiotic usage, supportive care with blood, platelet transfusion and growth factors, the hospital stay charges, the investigation charges and consultation fees. The data was obtained from computerized hospital information system. All patients were treated in Hepa-filtered BMT rooms in the 12 bedded BMT unit. Patients who expired before engraftment were excluded. The cost of outpatient follow-up or subsequent admissions was also excluded. The study also excluded the cost of procurement of matched unrelated donor harvest charges and the cost of HLA typing and donor assessment. Peripheral blood stem cell harvest was done in the blood bank by trained apheresis team; bone marrow harvest was done in the operation theater under general anesthesia. Transplant program employed a primary transplant team which conducted and monitored all pre-transplantation and post-transplantation care, supported by medical and pediatric intensivists. The study was approved by the Institutional Review Board and hospital’s Ethical committee.

### Conditioning regimen, GVHD prophylaxis, and supportive care

Conditioning before HSCT consisted of high-dose chemotherapy or reduced conditioning regimens with or without antithymocyte globulin. The commonly used regimens were busulfan/cyclophosphamide, fludarabine/cyclophosphamide/antithymocyte globulin, fludarabine/melphalan, thiotepa/triosulphan/fludarabine, melphalan and carmustine/etoposide/cytarabine/melphalan (Table 1). Conditioning regimen, graft source and graft versus host disease (GvHD) prophylaxis were protocol driven or based on the recommendation of the transplant team. The day of stem cell infusion was designated as day 0. For thalassemia major bone marrow was the source of stem cells and for leukemia and aplastic anemia, granulocyte colony stimulating factor (G-CSF)-mobilized peripheral blood stem cell from allogenic donor was the source of stem cells. For myeloma and lymphoma patients autologous stem cell harvest was done after G-CSF mobilization. Patients received standard anti-viral prophylaxis with acyclovir and Pneumocystis jiroveci prophylaxis with trimethoprim-sulfamethoxazole. Levofloxacin was used as bacterial prophylaxis if specified by protocols. Patients were treated with broad spectrum antibiotics at the time of their first neutropenic fever, and with antifungal agents as per institutional policy.

## Results

A total of 162 consecutive patients were evaluated for the cost of the procedure, focusing on the inpatient costs, till discharge from the hospital (Table 1). The median total cost of bone marrow transplantation was $ 16,650 (range $ 10,331–44,701). The median days of stay in the hospital were 33 days (range 17–56) (Table 2). Seven patients expired before engraftment and were excluded. The cost of management of acute gut GvHD grade II–IV was $ 11,600–25,500 extra. This cost study excluded the cost for treatment in those patients who developed GvHD after discharge from hospital and required readmission for GvHD treatment. Table 3 shows the distribution of the cost of stem cell transplantation.

## Discussion

HSCT is the cure for many hematological and non-hematological diseases; and in developing countries, where socio-economic status is a major limiting factor, the cost factor associated with BMT is an important issue. We retrospectively analyzed the costs of bone marrow transplantation in 162 consecutive patients transplanted in a tertiary care centre in northern India. The period of cost calculation was from the day of admission to the hospital for transplantation to the day of discharge. The median stay was 33 days (range 17–56 days). The median cost of autologous transplant was $ 12,500 (range $ 10,331–39,367) and the median cost of allogeneic transplant was $ 17,914 (range $ 10,832–44,701). The major cost was of the drugs (chemotherapeutic drugs and antimicrobials) and the blood products.

We also compared the cost of bone marrow transplants (BMT) done at our center with the cost of BMT in developed countries. Because of wide variations in the conditioning protocols and GVHD prophylaxis used, differences in supportive care practices, physician’s discretion in using the available resources and the different time periods of treatment follow-up, included in various studies, the cost factors are difficult to be compared. Still, when compared to the cost of the transplant in Europe and USA where it ranges from $30,000 to $88,000 for a single autologous transplantation to $200,000 or more for a matched unrelated myeloablative allogeneic procedure,^4^–^7^ the cost of the transplant in developing countries is much lower.^2^,^8^ In spite of this, many patients are not able to afford this due to low socio-economic condition in developing countries and lack of sufficient insurance companies and governmental support.

Cost of transplant also varies with the type of transplant (autologous, allogeneic), source of graft (sibling or matched unrelated), intensity of conditioning regimens used etc.^4^,^10^–^12^ No correlation has been found in the cost of transplant and patient’s age and sex, disease risk, or status.^5^,^9^ The cost of transplantation increases with the number of complications and duration of stay in the hospital.^5^,^7^,^12^ Cost of transplant increases in patients who develop grade III–IV acute GvHD,^4^,^6^,^7^ our patients who developed grade III–IV acute gut GvHD refractory to first line treatment had 2–3 times higher cost of the transplant.

The variation in the cost of the transplant is also directly related to the complications post transplant. These are a) infections (bacterial, fungal and viral), b) requirement of blood product transfusions, particularly because of the delay in platelet engraftment- requiring irradiated single donor platelets, c) Intensive care- patients requiring admission in medical intensive care units or ventilator support, d) onset of severe acute GvHD. Also, various infective and non-infective complications can develop later on, following the discharge from the hospital, and can increase the total cost of stem cell transplantation.^13^–^15^

In developing countries, the advantage of opting for transplant in patients with thalassemia major seems beneficial and much more cost effective than lifelong transfusion, chelation and investigation cost,^2^,^15^,^16^ with nearly equal success rates. Even in acute lymphoblastic leukemia, allogeneic transplant in CR-1 has been found to be cost effective compared to chemotherapy.^17^ The patients who deserve transplant should be considered for transplant early in the disease course to make it cost effective, particularly in developing countries, where mostly the cost is borne by the patients themselves, unlike in developed countries where the government or the insurance companies support.

The weakness of our study is that we have analyzed the cost of transplantation from the period of admission for transplantation till discharge after engraftment. The cost of successive admissions and the cost of GvHD prophylaxis and anti-microbial prophylaxis and the cost of regular out-patient follow-up and investigations were not included in the study. Moreover, the cost effectiveness was also not analyzed.

## Conclusion

The cost of bone marrow transplant till engraftment is much lesser in developing countries compared to developed countries with nearly equal success rate. In spite of this, many patients are not able to afford a much needed life saving procedure because of poor financial support. Further interventions to reduce the cost of the transplant to make it more affordable to the general population needs to be searched, considering the growing burden of patients with hematological diseases.

## Figures and Tables

**Table 1:** Transplant characteristic of the patients.

Total patients (n=162)	
Males	95 (58.6%)
Females	67 (41.4%)
Average Age (years, range)	27.8 (2–68)
Median hospital stay (days, range)	33 (17–56)
Type of transplant	
Autologous	38 (23.5%)
Allogeneic	124 (76.5%)
Sibling matched related	110 (88.7%)
Haplo-identical	6 (4.8%)
Matched unrelated	8 (6.4%)
Hematological diagnosis	
Thalassemia Major	51 (31.5%)
Acute Myeloid Leukemia	24 (14.8%)
Acute Lymphoblastic Leukemia	12 (7.4%)
Severe Aplastic Anemia	25 (15.4%)
Multiple Myeloma	22 (13.6%)
Non-Hodgkin Lymphoma	10 (6.17%)
Hodgkin Lymphoma	7 (4.3%)
Others	11 (6.8%)
Conditioning regimen	
Busulfan/Cyclophosphamide/±ATG	51 (31.5%)
Fludarabine/Cyclophosphamide/±ATG	27 (16.6%)
Fludarabine/Melphalan	20 (12.3%)
Treosulphan/Thiotepa/Fludarabine	27 (16.6%)
Melphalan	22 (13.6%)
Carmustine/Etoposide/Cytarabine/Melphalan	15 (9.3%)
Acute GVHD	
Grade I	11
Grade II–IV	12*

* Patients who developed GVHD before discharge; Patients who developed GVHD after discharge are not included.

**Table 2:** Cost of autologous and allogeneic bone marrow transplantation

Type of transplant	Number of patients (n=162)	Average Age (years, range)	Median Hospital stay (days, range)	Median total cost ($, range)
Autologous	38 (23.5%)	44.6 (7–63)	27 (19–39)	$ 12,500 ($ 10,331–39,367)
Allogeneic	124 (76.5%)	22.6 (2–68)	35 (17–56)	$ 17,914 ($ 10,832–44,701)

**Table 3:** The median distribution of charges and percentage contribution to total cost

Accommodation charges	$ 2754.3	16.6%
Blood product and transfusion charges	$ 2003.4	12.1%
Pharmacy charges	$ 4265.9	25.7%
Investigation charges	$ 1433.9	8.7%
Procedure charges including stem cell harvest and infusion	$ 2500.0	15%
Consultation fees	$ 1371.7	8.3%
Miscellaneous (including consumables, central catheter insertion, TPN etc)	$ 2320.4	13.6%
